# Emergency Department Cardiac Risk Stratification With High-Sensitivity vs Conventional Troponin HEART Pathway

**DOI:** 10.1001/jamanetworkopen.2023.48351

**Published:** 2023-12-19

**Authors:** Mackensie Yore, Adam Sharp, Yi-Lin Wu, Aniket Kawatkar, Ming-Sum Lee, Maros Ferencik, Rita Redberg, Ernest Shen, Chengyi Zheng, Benjamin Sun

**Affiliations:** 1 Department of Emergency Medicine, Veterans Affairs/University of California Los Angeles National Clinician Scholars Program, Los Angeles; 2 Clinical Science Department, Kaiser Permanente Bernard J. Tyson School of Medicine, Pasadena, California; 3 Department of Research and Evaluation, Kaiser Permanente Southern California, Pasadena; 4 Department of Cardiology, Kaiser Permanente Los Angeles Medical Center, Los Angeles, California; 5 Knight Cardiovascular Institute, Oregon Health & Science University, Portland; 6 Division of Cardiology, University of California, San Francisco; 7 Philip R. Lee Institute for Health Policy Studies, University of California, San Franciscio; 8 Department of Emergency Medicine, Leonard Davis Institute of Health Economics, University of Pennsylvania, Philadelphia

## Abstract

**Importance:**

Patients presenting to the emergency department with chest pain are routinely risk stratified for major adverse cardiac events using the HEART (History, Electrocardiogram, Age, Risk factors, and Troponin) score pathway, which incorporates clinical features, risk factors, electrocardiography findings, and initial serum troponin testing. A new HEART pathway incorporating high-sensitivity troponin level may improve risk stratification among patients with possible acute myocardial infarction (AMI).

**Objective:**

To compare health outcomes and resource use among emergency department patients undergoing cardiac risk stratification with a HEART pathway using conventional vs high-sensitivity serum troponin.

**Design, Setting, and Participants:**

This multicenter pre-post cohort study was conducted between January 1 and September 6, 2021, at 16 Kaiser Permanente Southern California hospitals during uptake of a high-sensitivity serum troponin assay and included 17 384 adult patients who presented to an emergency department with chest pain and were risk stratified with a HEART pathway based on conventional troponin or high-sensitivity troponin.

**Exposures:**

A HEART pathway incorporating either conventional or high-sensitivity serum troponin was used to stratify study groups for risk of major adverse cardiac events within 30 days.

**Main Outcomes and Measures:**

The primary outcome was detection of AMI in the emergency department and within 30 days.

**Results:**

Of the 17 384 patients (median age, 58 years [IQR, 45-69 years]; 9767 women [56.2%]), 12 440 (71.6%) were risk stratified with a HEART pathway based on conventional troponin, and 4944 (28.4%) were risk stratified with a HEART pathway based on high-sensitivity troponin. Detection of AMI within 30 days was higher for the high-sensitivity troponin group than the conventional troponin group (288 [5.8%] vs 545 [4.4%]; *P* < .001), while the 30-day all-cause mortality rate was unchanged (16 [0.3%] vs 50 [0.4%]; *P* = .50). In the emergency department, 228 of 4944 patients (4.6%) in the high-sensitivity troponin group received a diagnosis of AMI compared with 251 of 12 440 patients (2.0%) in the conventional troponin group (*P* < .001). Among those who did not receive a diagnosis of AMI in the emergency department, an additional 60 patients (1.2%) in the high-sensitivity troponin group and 294 (2.4%) in the conventional troponin group (*P* < .001) received a diagnosis within 30 days. Patients in the high-sensitivity troponin group had lower rates of health care use compared with the conventional troponin group, including admission (605 [12.2%] vs 1862 [15.0%]; *P* < .001), stress testing within 7 days (506 [10.2%] vs 1591 [12.8%]; *P* < .001), and coronary revascularization within 30 days (51 [1.0%] vs 244 [2.0%]; *P* < .001).

**Conclusions and Relevance:**

This multicenter pre-post cohort study suggests that a new HEART pathway incorporating high-sensitivity troponin may improve detection of AMI and decrease resource use among emergency department patients with chest pain.

## Key Points

**Question:**

Are 30-day health outcomes and health service use different among emergency department (ED) patients undergoing cardiac risk stratification with a HEART pathway using conventional vs high-sensitivity troponin level?

**Findings:**

In a multicenter cohort study of 17 384 ED patients with chest pain risk stratified with a HEART pathway incorporating conventional vs high-sensitivity troponin level, the high-sensitivity troponin group showed a higher rate of acute myocardial infarction (AMI) diagnosis during the ED visit and a lower rate of AMI diagnosis within 30 days after the visit, along with lower rates of admission, stress testing, and revascularization. Mortality was rare and similar across groups.

**Meaning:**

This study suggests that a high-sensitivity troponin HEART pathway may be associated with earlier detection of AMI and improved resource use.

## Teaser

This cohort study compares health outcomes and resource use among emergency department patients undergoing cardiac risk stratification with a HEART (History, Electrocardiogram, Age, Risk Factors, and Troponin) pathway using conventional vs high-sensitivity serum troponin.

## Introduction

Chest pain, a classic symptom of acute myocardial infarction (AMI), is the second most common reason for emergency department (ED) visits in the US.^1^ Differentiating potentially life-threatening causes from other causes of chest pain is a common, high-stakes task. Historically, failure to detect AMI in the ED has been associated with increased mortality risk.^2^ Consequently, clinicians frequently overuse advanced testing for patients with chest pain, resulting in unnecessary hospitalizations, billions of dollars in avoidable annual costs, and iatrogenic harm without clinical benefits.^3,4^

Emergency departments in the US commonly use the HEART (History, Electrocardiogram, Age, Risk factors, and Troponin) score for cardiac risk stratification, combining patient history, risk factors, electrocardiography findings, and serum troponin.^5^ Some hospitals formally incorporate HEART scores into diagnostic and management pathways for patients presenting with chest pain. In 2017, the US Food and Drug Administration approved a higher-sensitivity troponin (hsTn) assay capable of detecting cardiac-specific serum troponin at lower levels than conventional troponin (cTn),^6^ potentially enabling earlier rule-in or rule-out of AMI. Previous studies on the association of hsTn with patient care are mostly from outside the US and present equivocal results on clinical outcomes and service use^7,8,9,10,11,12^; to our knowledge, little is known about the association of switching to an hsTn-based HEART pathway with patient outcomes and service use in clinical practice in the US. We conducted the first large, US multicenter study comparing health outcomes and care use among adults presenting to the ED with potential AMI before and after implementation of a new hsTn-based HEART pathway.

## Methods

### Study Design and Participants

Kaiser Permanente Southern California (KPSC) serves 4.6 million Kaiser members across 21 hospitals; KPSC data include outcomes and service use at KPSC and non-KPSC facilities and deaths anywhere. In 2021, a total of 16 KPSC EDs phased in hsTn, providing an opportunity to examine differences in 30-day health outcomes and health care use among patients receiving risk stratification with the cTn HEART pathway (cTn group) vs the hsTn HEART pathway (hsTn group) in a multicenter pre-post cohort study. Our sample included adults (≥18 years) presenting to the ED from January 1 to September 6, 2021, with chest pain who were risk stratified with a HEART pathway. We excluded transferred patients, those receiving hospice care, anyone with a COVID-19 diagnosis or positive test result during or within 1 month prior to the index ED visit, and anyone with do-not-resuscitate status. All research procedures were conducted in accordance with the KPSC institutional review board, which granted a waiver of informed consent for this study because this was a quality improvement initiative and it was not feasible to collect informed consent on all eligible patients receiving care for suspected acute coronary syndrome. This study followed the Strengthening the Reporting of Observational Studies in Epidemiology (STROBE) reporting guideline.

### Laboratory Troponin Testing and HEART Pathways

At least 2 months after the study’s start and 5 weeks before its end, each hospital completely transitioned from cTn (AccuTnI+3 assay; Beckman Coulter Inc) and the conventional HEART pathway (Figure, A) to hsTn (Access hsTnI assay; Beckman Coulter Inc) and a new HEART pathway (Figure, B) developed by KPSC emergency medicine, cardiology, and hospitalist physicians with Beckman Coulter Diagnostics consultants that builds on the RAPID-TnT (Rapid Assessment of Possible Acute Coronary Syndrome in the Emergency Department With High-Sensitivity Troponin T) trial,^11^ incorporating Beckman Coulter validation data and previously reported sex-specific AMI rule-in or rule-out thresholds.^13,14^ The main differences between the HEART pathways are (1) the new HEART pathway includes a branch point for presentation within or after 3 hours after symptom onset, (2) AMI rule-in hsTn levels for late-presenting patients, (3) incorporation of repeat troponin values 2 hours apart, and (4) updated numerical scores given the increased sensitivity of hsTn. Ordering a cTn or hsTn test auto-prompted clinicians to document answers to HEART score questions to generate the HEART score used in the relevant HEART pathway. Cardiac risk stratification with either the cTn- or hsTn-based HEART pathway was considered the intervention in this study.

**Figure.**
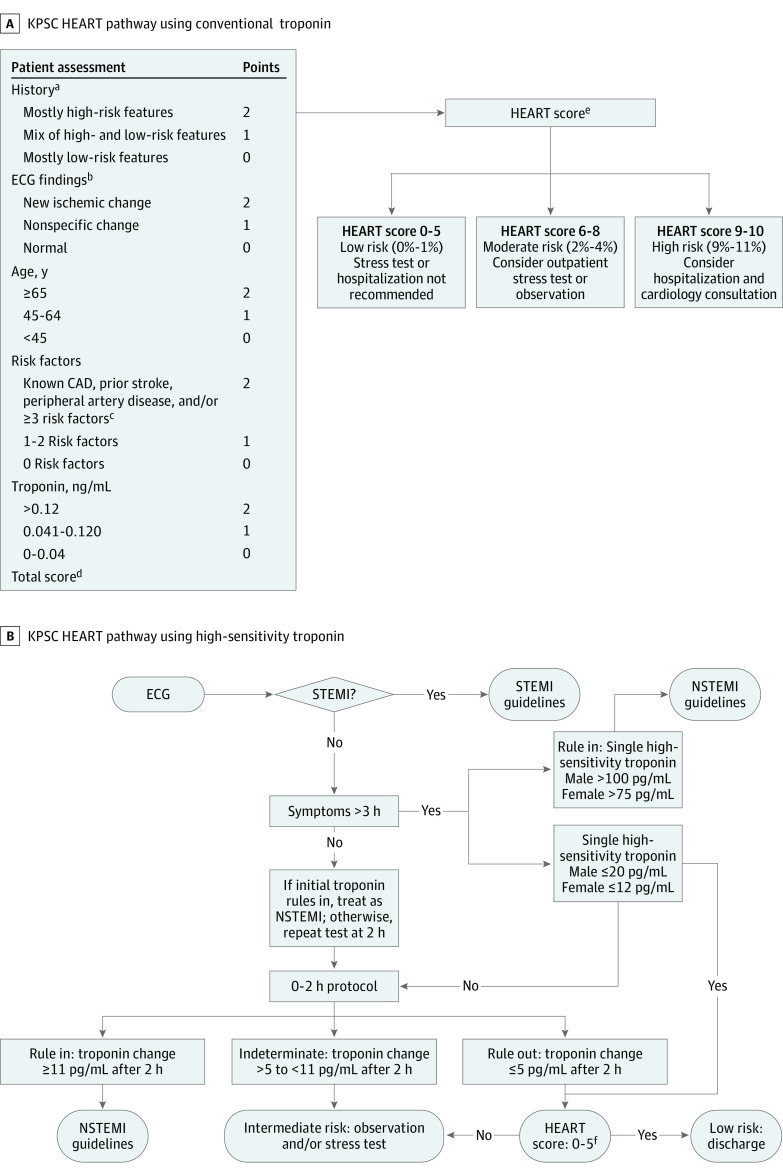
Kaiser Permanente Southern California (KPSC) HEART Pathway Using Conventional Troponin and High-Sensitivity Troponin The main differences between the HEART pathways are as follows: (1) the high-sensitivity troponin HEART pathway includes a branch point for presentation within or after 3 hours after symptom onset, (2) acute myocardial infarction rule-in high-sensitivity troponin levels for late-presenting patients, (3) incorporation of repeat troponin values 2 hours apart, and (4) updated numerical scores given high-sensitivity troponin’s increased sensitivity. CAD indicates coronary artery disease; ECG, electrocardiography; NSTEMI, non–ST-segment elevation myocardial infarction; and STEMI, ST-segment elevation myocardial infarction. SI conversion factor: To convert troponin to micrograms per liter, multiply by 0.001. ^a^High-risk features: middle or left-sided pain, heavy chest pain, diaphoresis, relief of symptoms by sublingual nitrates, nausea or vomiting, radiating pain, and exertional pain. Low-risk features: well-localized pain, sharp pain, nonexertional pain, no diaphoresis, and no nausea or vomiting. ^b^New ischemic changes: ischemic ST-segment depression and new ischemic T-wave changes. Nonspecific changes: repolarization abnormality, nonspecific T-wave changes, bundle-branch block, digoxin effect, pacemaker rhythms, left ventricular hypertrophy, early repolarization, and nonspecific ST-segment depression or elevation. ^c^Risk factors: obesity (body mass index [calculated as weight in kilograms divided by height in meters squared] ≥30), current or recent (≤90 days) smoker, currently treated diabetes, family history of CAD (first-degree relative with CAD at younger than 55 years), diagnosed and/or treated hypertension, and hypercholesterolemia. ^d^Total score (range, 0-10) is sum of the points (0, 1, or 2) assigned in each category (eg, History, ECG findings). ^e^Percentages refer to 30-day mortality or acute myocardial infarction risk. ^f^HEART score calculated as in A with substitution of assigned point values for high-sensitivity troponin levels by sex (women, >36 = 2 points, 12-36 = 1 point, <12 = 0 points; men, >60 = 2 points, 20-60 = 1 point, <20 = 0 points).

### Outcomes

Primary outcomes were all-cause mortality and AMI detection, both during the ED visit and within 30 days after. We ascertained AMI outcomes from *International Statistical Classification of Diseases and Related Health Problems, Tenth Revision* codes in billing documentation.^15^ Secondary outcomes were hospital admission, stress testing within 7 days, and coronary revascularization within 30 days.

### Statistical Analysis

We compared baseline characteristics and outcomes between the cTn and hsTn groups using 2-sided Wilcoxon rank sum tests (continuous variables) and χ^2^ tests (categorical variables) with SAS, version 9.4 (SAS Institute Inc), with a significance level of *P* < .05. Supplemental analyses compared baseline characteristics and outcomes of patients with and patients without documented HEART scores using proportions testing (categorical variables) and *t* tests (continuous variables) with STATA SE, version 14.1 (StataCorp LP), with a significance level of *P* < .05.

To account for potential differences in baseline characteristics between groups risk stratified with cTn vs hsTn HEART pathway, we used multivariable logistic regression to evaluate the odds ratio of AMI diagnosis after ED discharge but within 30 days of the index ED visit associated with the exposure of the cTn- vs hsTn-based HEART pathway for cardiac risk stratification. The regression model adjusted for the following potential confounders: age, sex, race (Asian, Black, or other), Hispanic ethnicity, HEART score category (low, moderate, or high risk), obesity (body mass index ≥30.0 [calculated as weight in kilograms divided by height in meters squared]), prior diagnosis of coronary artery disease or stroke, prior coronary revascularization, family history of coronary artery disease, and Elixhauser comorbidity index.^16^ Race and ethnicity were included as proxies of social determinants of health and as potential confounding variables. Race and ethnicity were categorized as per National Institutes of Health standards for classification and obtained from the health plan’s administrative records.

## Results

### Cohort Characteristics

Of the total 28 258 adults who presented to a KPSC ED for chest pain and underwent troponin testing, 17 384 (median age, 58 years [IQR, 45-69 years]; 9767 women [56.2%]) were included in the final cohort, while 10 874 patients (38.5%) were excluded due to missing HEART scores (n = 7963), positive COVID-19 test result or COVID-19 diagnosis (n = 1443), do-not-resuscitate status (n = 1196), transfer from another hospital (n = 270), or hospice enrollment (n = 2). When compared as a group with patients with HEART scores, patients with missing HEART scores were younger (mean [SD] age, 55.6 [17.6] years vs 56.6 [16.7] years; *P* < .001), had a higher mean (SD) Elixhauser comorbidity index score (3.4 [2.9] vs 3.3 [2.8]; *P* = .009), were less likely to have hypertension (48.9% [3890 of 7963] vs 50.7% [8814 of 17 384]; *P* = .008), and were more likely to have congestive heart failure (721 of 7963 [9.1%] vs 1447 of 17 384 [8.3%]; *P* = .04) (eTables 1 and 2 in Supplement 1). The fraction of patients risk stratified using the hsTn-based HEART pathway was not significantly different between patients with and patients without documented HEART scores (28.4% [4944 of 17 384] vs 29.2% [2324 of 7963]; *P* = .19).

Of the 17 384 patients included in the final cohort (Table 1), 12 440 (71.6%) were tested with cTn and 4944 (28.4%) with hsTn. Patients in the cTn group were more likely than those in the hsTn group to have a higher mean (SD) Elixhauser comorbidity index (3.3 [2.8] vs 3.2 [2.7]; *P* = .04), diabetes (3439 [27.6%] vs 1243 [25.1%]; *P* < .001), and high cholesterol (7458 [60.0%] vs 2782 [56.3%]; *P* < .001) and to have previously undergone coronary revascularization (159 [1.3%] vs 36 [0.7%]; *P* < .001) and invasive coronary angiography (291 [2.3%] vs 67 [1.4%]; *P* < .001). Kidney failure was more common in the hsTn group than in the cTn group (723 [14.6%] vs 1674 [13.5%]; *P* = .04).

**Table 1.** Baseline Patient Characteristics

Characteristic	Patients, No. (%)		*P* value
	Conventional troponin (n = 12 440)	High-sensitivity troponin (n = 4944)	
Age, mean (SD), y	56.6 (16.5)	56.5 (17.3)	.72
Female	6986 (56.2)	2781 (56.3)	.91
Hispanic ethnicity	5473 (44.0)	1794 (36.3)	<.001
Race			
Alaska Native or Pacific Islander	220 (1.8)	84 (1.7)	<.001
Asian	1334 (10.7)	581 (11.8)	
Black	1709 (13.7)	859 (17.4)	
White	6837 (55.0)	2656 (53.7)	
Other	2340 (18.8)	764 (15.5)	
Smoking behavior			
Never	7613 (61.2)	3053 (61.8)	.13
Quit	56 (0.5)	30 (0.6)	
Passive	3235 (26.0)	1215 (24.6)	
Active	629 (5.1)	278 (5.6)	
Missing	907 (7.3)	368 (7.4)	
Family history of coronary artery disease	4598 (37.0)	1795 (36.3)	.42
Elixhauser comorbidity index, mean (SD)	3.3 (2.8)	3.2 (2.7)	.04
Hypertension	6356 (51.1)	2458 (49.7)	.10
Diabetes	3439 (27.6)	1243 (25.1)	<.001
High cholesterol	7458 (60.0)	2782 (56.3)	<.001
Congestive heart failure	1039 (8.4)	408 (8.3)	.83
Kidney failure	1674 (13.5)	723 (14.6)	.04
Coronary artery disease	2266 (18.2)	893 (18.1)	.81
Prior coronary revascularization	159 (1.3)	36 (0.7)	.002^a^
CABG	39 (0.3)	9 (0.2)	.15^a^
PTCA	126 (1.0)	28 (0.6)	.004^a^
Prior invasive angiogram	291 (2.3)	67 (1.4)	<.001^a^

Abbreviations: CABG, coronary artery bypass graft; PTCA, percutaneous transluminal coronary angioplasty.

^a^ Fisher exact test.

### Health Outcomes and Health Care Use

Table 2 provides data on health outcomes and health care use for both groups. A total of 288 of 4944 patients (5.8%) receiving hsTn received a diagnosis of AMI within 30 days compared with 545 of 12 440 (4.4%) receiving cTn (*P* < .001), while the 30-day all-cause mortality rate was unchanged (16 of 4944 [0.3%] in the hsTn group vs 50 of 12 440 [0.4%] in the cTn group; *P* = .50). Among those who received a diagnosis of AMI within 30 days of ED presentation, 79.1% (228 of 288) in the hsTn group received a diagnosis during their initial ED visit compared with 46.1% (251 of 545) in the cTn group (*P* < .001). There were 60 of 4944 additional patients (1.2%) in the hsTn group who received a diagnosis of AMI within 30 days after the index ED visit compared with 294 of 12 440 (2.4%) in the cTn group (*P* < .001). In the ED and within 30 days, death was rare, with no significant difference detected between groups.

**Table 2.** Health Outcomes and Health Care Use

Outcome	Patients, No. (%)		*P* value
	Conventional troponin (n = 12 440)	High-sensitivity troponin (n = 4944)	
Cumulative outcomes within 30 d			
Death^a^	50 (0.4)	16 (0.3)	.50
AMI^a^^,^^b^	545 (4.4)	288 (5.8)	<.001
Outcomes in the ED			
Death^a^	1 (0.01)	1 (0.02)	.49
AMI^a^^,^^b^	251 (2.0)	228 (4.6)	<.001
Outcomes within 30 d, excluding ED outcomes			
Death^a^	49 (0.4)	15 (0.3)	.41
AMI^a^^,^^b^	294 (2.4)	60 (1.2)	<.001
Admission to hospital, observation, or operating room	1862 (15.0)	605 (12.2)	<.001
Stress test within 7 d^c^	1591 (12.8)	506 (10.2)	<.001
Coronary revascularization within 30 d^a^^,^^c^	244 (2.0)	51 (1.0)	<.001
CABG^a^	72 (0.6)	21 (0.4)	.25
PTCA^a^	175 (1.4)	31 (0.6)	<.001
HEART score (risk groups)^a^			
≥9 (High)	75 (0.6)	40 (0.8)	.27
6-8 (Intermediate)	1378 (11.1)	533 (10.8)	
≤5 (Low)	10987 (88.3)	4371 (88.4)	

Abbreviations: AMI, acute myocardial infarction; CABG, coronary artery bypass graft; ED, emergency department; PTCA, percutaneous transluminal coronary angioplasty.

^a^ Fisher exact test.

^b^ Defined by diagnosis code and troponin value.

^c^ From ED arrival.

The rate of admission from the ED was higher in the cTn group compared with the hsTn group (1862 of 12 440 [15.0%] vs 605 of 4944 [12.2%]; *P* < .001) (Table 2). In the cTn group, the rate of stress testing within 7 days was also higher compared with the hsTn group (1591 of 12 440 [12.8%] vs 506 of 4944 [10.2%]; *P* < .001), and coronary revascularization within 30 days was more common (244 of 12 440 [2.0%] vs 51 of 4944 [1.0%]; *P* < .001).

Compared with all patients with documented HEART scores, patients who received troponin testing but did not receive a documented HEART score due to 1 or more missing answers to HEART score questions had a significantly greater all-cause mortality rate (46 of 7963 [0.6%] vs 66 of 17 384 [0.4%]; *P* = .03), AMI diagnosis within 30 days (464 of 7963 [5.8%] vs 833 of 17 384 [4.8%]; *P* < .001), and coronary revascularization within 30 days (168 of 7963 [2.1%] vs 295 of 17 384 [1.7%]; *P* = .03) (eTable 1 in Supplement 1).

### Adjusted Mean Probability of AMI After the Index ED Visit and Within 30 Days

The adjusted odds ratio of AMI diagnosis after the index ED visit and within 30 days with risk stratification using the hsTn-based HEART pathway vs the cTn-based pathway was 0.54 (95% CI, 0.40-0.72) (eTable 3 in Supplement 1). The difference in marginal mean probability of AMI within 30 days between patients stratified by the hsTn pathway (0.014) and patients stratified by the cTn pathway (0.024) was −0.010 (95% CI, −0.005 to −0.014), suggesting reduction of 1 AMI diagnosis within 30 days after the index visit for every 100 patients stratified with the hsTn vs cTn pathway.

## Discussion

Compared with the cTn pathway, we found a small statistically significant increase in the percentage of patients risk stratified with the hsTn pathway who received a diagnosis of AMI at the index ED visit, coupled with a small statistically significant decrease in the percentage of patients who received a diagnosis of AMI in the 30 days after the index visit. Rates of admission, stress testing, and coronary revascularization were lower after implementation of the hsTn pathway; these reductions were small, although given the number of ED visits for chest pain, they may be meaningful. Our results suggest a useful role for an hsTn pathway for helping assess which patients have a lower risk of near-term major adverse cardiac events, allowing them to be safely managed without hospital admission or invasive testing and also perhaps allowing for earlier diagnosis of patients with AMI (eg, during the ED visit). Further analyses are needed to determine whether, and to what extent, the small decreases in health care use seen using the more involved hsTn HEART pathway translate to meaningful cost savings with more widespread implementation. Although we found no difference in mortality between the hsTn and cTn groups, death was a rare event in both groups, and larger studies with a longer follow-up period are needed to further evaluate potential differences in mortality and morbidity. Furthermore, to address the question of the specificity of hsTn for detecting true AMI, future studies tracking individual patient results from subsequent tests (eg, angiography) after an elevated hsTn level could further differentiate the true- vs false-positive rate of AMI detection with hsTn.

Nearly one-third of patients (7963 of 28 258 [28.2%]) received troponin testing but were not assigned HEART scores and were therefore excluded from this analysis. Similar fractions of patients assigned to the cTn and hsTn pathways lacked HEART scores, suggesting that HEART scores were missing due to patient characteristics or clinician decisions rather than differences in pathway complexity. We found that the group of patients lacking HEART scores had increased cardiovascular risk (eg, higher mean Elixhauser comorbidity index score and higher prevalence of congestive heart failure) and poorer health outcomes (eg, greater rates of all-cause mortality, AMI diagnosis within 30 days, and coronary revascularization within 30 days). Therefore, it is possible that clinicians sometimes dismissed the HEART score–generating prompts for patients with many known cardiovascular risk factors and managed these patients as if they had high-risk scores. Overall, our finding that patients with missing HEART scores had poorer cardiovascular health and worse health outcomes reenforces the usefulness of the HEART pathway for patients at lower risk, for whom clinicians may rely more on a decision-making pathway than they would for patients with obvious AMI or with sufficiently high risk for AMI that they require admission regardless of HEART score.

There was 1 previous US study comparing the use of hsTn and cTn. In a small secondary subset analysis of patients enrolled in the HEART pathway randomized clinical trial who were risk stratified with hsTn rather than cTn, there was a similar detection of major adverse cardiac events in both groups^12^; however, the study’s small size limited its ability to have detected any differences. European studies have shown no change in mortality or AMI when switching to hsTn,^11,17^ while our study, embedding hsTn within a modified version of a widely used diagnostic algorithm, showed an increase in sensitivity for detecting AMI. Furthermore, our study showed a decrease in the admission rate from 15.0% to 12.2%, an 18.7% relative decrease vs other studies showing a 0% to 13% decrease.^11,17^ Given the potential benefits of hsTn to the health system demonstrated in our study, including fewer missed AMI diagnoses in the ED and decreased resource use, an important next step will be to test the replication in other US health systems and to examine outcomes of hsTn implementation specific to the ED (eg, length of ED stay) and other areas within the health care system.

### Limitations

This study has some limitations. First, KPSC is a large, integrated health care system serving patients with health insurance, where prompt outpatient follow-up and testing may be challenging to replicate in other settings. Second, while a 30-day follow-up interval shows outcomes useful to ED clinicians, 1-year data would allow for a more comprehensive evaluation of the public health effect of hsTn. Third, this study occurred during the COVID-19 pandemic; although we excluded patients with active COVID-19, health and/or health care use outcomes may reflect increased ED discharge and decreased follow-up testing to protect patients from COVID-19.

## Conclusions

In this multicenter pre-post cohort study of an hsTn- vs cTn-based HEART pathway as part of ED evaluation for AMI, the hsTn HEART pathway was associated with higher rates of ED AMI diagnoses and lower rates of AMI diagnoses after the index ED visit and within 30 days. In addition, we found less health service use for the hsTn group compared with the cTn group. An hsTn algorithm may improve the ED evaluation of AMI, both catching AMI earlier and mitigating unnecessary admission and advanced testing.

## Notes

Supplement 1. **eTable 1.** Characteristics of KP Members With and Without HEART Scores **eTable 2.** Characteristics of KP Members With and Without HEART Scores, Stratified by cTn and hsTn HEART Pathway **eTable 3.** Odds Ratios From Multivariable Logistic Regression Model Examining Factors Associated With Risk of AMI Diagnosis Within 30 Days, Excluding Index ED Visit

Supplement 2.
Data Sharing Statement
