# Supplementary material for: Emergency Department Cardiac Risk Stratification With High-Sensitivity vs Conventional Troponin HEART Pathway
Source: JAMA Netw Open. 2023 Dec 19;6(12):e2348351. doi: 10.1001/jamanetworkopen.2023.48351 (PMC10731477; doi:10.1001/jamanetworkopen.2023.48351)
Supplement: Supplement 1. — eTable 1. Characteristics of KP Members With and Without HEART Scores eTable 2. Characteristics of KP Members With and Without HEART Scores, Stratified by cTn and hsTn HEART Pathway eTable 3. Odds Ratios From Multivariable Logistic Regression Model Examining Factors Associated With Risk of AMI Diagnosis Within 30 Days, Excluding Index ED Visit [file jamanetwopen-e2348351-s001.pdf]

Supplementary Online Content

Yore M, Sharp A, Wu YL, et al. Emergency department cardiac risk stratification with high-sensitivity vs conventional troponin HEART pathway. *JAMA Netw Open.* 2023;6(12):e2348351. doi:10.1001/jamanetworkopen.2023.48351

eTable 1. Characteristics of KP Members With and Without HEART Scores
eTable 2. Characteristics of KP Members With and Without HEART Scores, Stratified by cTn and hsTn HEART Pathway
eTable 3. Odds Ratios From Multivariable Logistic Regression Model Examining Factors Associated With Risk of AMI Diagnosis Within 30 Days, Excluding Index ED Visit

This supplementary material has been provided by the authors to give readers additional information about their work.

© 2023 Yore M et al. *JAMA Network Open.*

eTable 1. Characteristics of KP Members With and Without HEART Scores

Characteristic	Including HEART scores	Missing HEART scores	p
N	17,384	7,963	
hsTn pathway (%)	4944 (28.4)	2324 (29.1)	0.19
Mean age (SD)	56.6 (16.7)	55.6 (17.58)	<0.001
Female, N (%)	9767 (56.2)	4534 (56.9)	0.30
Hispanic Ethnicity, N (%)	7267 (41.8)	3360 (42.2)	0.55
Elixhauser Comorbidities, Mean (SD)	3.3 (2.78)	3.4 (2.94)	0.009
Hypertension	8814 (50.7)	3890 (48.9)	0.008
Diabetes	4682 (26.9)	2139 (26.9)	1.00
Congestive Heart Failure	1447 (8.3)	721 (9.1)	0.03
Renal Failure	2397 (13.8)	1167 (14.7)	0.06
Prior Coronary Revascularization, N (%)	195 (1.1)	82 (1)	0.47
CABG, N (%)	48 (0.3)	26 (0.3)	1.00
PTCA, N (%)	154 (0.9)	57 (0.7)	0.10
AMI or Died within 30 Days, N (%)[a]	871 (5)	492 (6.2)	<0.001
Death, N (%)	66 (0.4)	46 (0.6)	0.03
AMI, N (%)[b]	833 (4.8)	464 (5.8)	0.001
Coronary revascularization within 30 Days, N (%)[a]	295 (1.7)	168 (2.1)	0.03
CABG, N (%)	93 (0.5)	48 (0.6)	0.31
PTCA, N (%)	206 (1.2)	122 (1.5)	0.05

Proportions test was used for categorical variables, and t test was used for continuous variables.

[a]from ED arrival

[b]defined by diagnosis code and troponin value

© 2023 Yore M et al. *JAMA Network Open.*

**eTable 2.** Characteristics of KP Members With and Without HEART Scores, Stratified by cTn and hsTn HEART Pathway						
	**Including HEART scores (N = 7384)**			**Missing HEART scores (N = 7963)**		
**Characteristic**	**cTn**	**hsTn**	**p**	**cTn**	**hsTn**	**p**
Mean age (SD)	56.6 (16.47)	56.5 (17.27)	0.72	55.7 (17.39)	55.2 (18.03)	0.27
Female, N (%)	6986 (56.2%)	2781 (56.3%)	0.91	3206 (56.9%)	1328 (57.1%)	0.79
Hispanic Ethnicity, N (%)	5473 (44%)	1794 (36.3%)	<0.001	2490 (44.2%)	870 (37.4%)	<0.001
Elixhauser Comorbidities, Mean (SD)	3.3 (2.80)	3.2 (2.73)	0.04	3.5 (2.99)	3.3 (2.82)	0.06
Hypertension	6356 (51.1%)	2458 (49.7%)	0.10	2799 (49.6%)	1091 (46.9%)	0.03
Diabetes	3439 (27.6%)	1243 (25.1%)	<0.001	1543 (27.4%)	596 (25.6%)	0.12
Congestive Heart Failure	1039 (8.4%)	408 (8.3%)	0.83	528 (9.4%)	193 (8.3%)	0.13
Renal Failure	1674 (13.5%)	723 (14.6%)	0.04	827 (14.7%)	340 (14.6%)	0.97
Prior Coronary Revascularization, N (%)[a]	159 (1.3%)	36 (0.7%)	0.002	62 (1.1%)	20 (0.9%)	0.39
CABG, N (%)[a]	39 (0.3%)	9 (0.2%)	0.15	19 (0.3%)	7 (0.3%)	1.00
PTCA, N (%)[a]	126 (1%)	28 (0.6%)	0.004	43 (0.8%)	14 (0.6%)	0.56
AMI or Died within 30 Days, N (%)[ab]	575 (4.6%)	296 (6%)	<0.001	298 (5.3%)	194 (8.3%)	<0.001
Death, N (%)[a]	50 (0.4%)	16 (0.3%)	0.50	30 (0.5%)	16 (0.7%)	0.42
AMI, N (%)[ac]	545 (4.4%)	288 (5.8%)	<0.001	282 (5%)	182 (7.8%)	<0.001
Coronary revascularization within 30 Days, N (%)[ab]	244 (2%)	51 (1%)	<0.001	126 (2.2%)	42 (1.8%)	0.26
CABG, N (%)[a]	72 (0.6%)	21 (0.4%)	0.25	35 (0.6%)	13 (0.6%)	0.87
PTCA, N (%)[a]	175 (1.4%)	31 (0.6%)	<0.001	93 (1.6%)	29 (1.2%)	0.19
Chi-square test was used for categorical variables, and Wilcoxon test was used for continuous variables.						
[a]Fisher's Exact Test						
[b]from ED arrival						
[c]defined by diagnosis code and troponin value						

© 2023 Yore M et al. *JAMA Network Open.*

eTable 3. Odds Ratios From Multivariable Logistic Regression Model Examining Factors Associated With Risk of AMI Diagnosis Within 30 Days, Excluding Index ED Visit

	**Adjusted OR**	**(OR 95% CI)**
hsTn pathway[a]	0.54	(0.4, 0.72)
Age	1.00	(0.99, 1.01)
Female	0.75	(0.59, 0.95)
Race		
Asian	1.47	(1.04, 2.08)
Black	1.21	(0.86, 1.69)
Others	1.00	(0.71, 1.39)
Hispanic ethnicity	1.03	(0.79, 1.35)
Heart score category		
Moderate risk	4.10	(2.63, 6.39)
High risk	21.83	(13.03, 36.56)
BMI 30.0 or higher	1.11	(0.88, 1.4)
Prior diagnosis of coronary artery disease	0.25	(0.18, 0.34)
Prior diagnosis of stroke	1.05	(0.6, 1.86)
Prior coronary revascularization	1.08	(0.71, 1.64)
Family history of coronary artery disease	0.98	(0.78, 1.24)
Elixhauser comorbidities	0.96	(0.92, 1.01)

[a]Risk-stratified using hsTn-based HEART pathway

© 2023 Yore M et al. *JAMA Network Open.*
