# Supplementary material for: Emergency Department Cardiac Risk Stratification With High-Sensitivity vs Conventional Troponin HEART Pathway
Source: JAMA Netw Open. 2023 Dec 19;6(12):e2348351. doi: 10.1001/jamanetworkopen.2023.48351 (PMC10731477; doi:10.1001/jamanetworkopen.2023.48351)
Supplement: Supplement 2. — Data Sharing Statement [file jamanetwopen-e2348351-s002.pdf]

Data Sharing Statement

Yore. Emergency Department Cardiac Risk Stratification With High-Sensitivity vs Conventional Troponin HEART Pathway. *JAMA Netw Open*. Published December 19, 2023. doi:10.1001/jamanetworkopen.2023.48351

Data
Data available: No
